# Pituitary Actions of EGF on Gonadotropins, Growth Hormone, Prolactin and Somatolactins in Grass Carp

**DOI:** 10.3390/biology9090279

**Published:** 2020-09-08

**Authors:** Qiongyao Hu, Qinbo Qin, Shaohua Xu, Lingling Zhou, Chuanhui Xia, Xuetao Shi, Huiying Zhang, Jingyi Jia, Cheng Ye, Zhan Yin, Guangfu Hu

**Affiliations:** 1 College of Fisheries, Huazhong Agricultural University, Wuhan 430070, China; HQY960819@163.com (Q.H.); xsh2018308@163.com (S.X.); llz9872@163.com (L.Z.); 13656721403@163.com (C.X.); sxt0902@163.com (X.S.); zhy_meme@163.com (H.Z.); Jiajy94@163.com (J.J.); yechenging@163.com (C.Y.); 2 State Key Laboratory of Developmental Biology of Freshwater Fish, Hunan Normal University, Changsha 410081, China; qinqinbo66@126.com; 3 State Key Laboratory of Freshwater Ecology and Biotechnology, Institute of Hydrobiology, Chinese Academy of Sciences, Wuhan 430072, China

**Keywords:** EGF, ErbB1, prolactin, growth hormone, LHβ, NKB

## Abstract

**Simple Summary:**

In mammals, the functions of epidermal growth factor (EGF) have been widely studied. However, little is known about the pituitary actions of EGF in teleost. Using primary cultured grass carp pituitary cells as model, we found that EGF could reduce pituitary luteinizing hormone β (LHβ) mRNA expression, but induce pituitary growth hormone (GH), prolactin (PRL) and somatolactins (SL) mRNA expression. Furthermore, we also found that NKB could suppress EGF-induced PRL mRNA expression in grass carp pituitary cells. These results suggested that EGF could directly regulate pituitary hormones expression in teleost.

**Abstract:**

In mammals, epidermal growth factor (EGF) plays a vital role in both pituitary physiology and pathology. However, the functional role of EGF in the regulation of pituitary hormones has rarely reported in teleost. In our study, using primary cultured grass carp pituitary cells as an in vitro model, we examined the effects of EGF on pituitary hormone secretion and gene expression as well as the post-receptor signaling mechanisms involved. Firstly, we found that EGF significantly reduced luteinizing hormone (LHβ) mRNA expression via ErbB1 coupled to ERK1/2 pathway, but had no effect on LH release in grass carp pituitary cells. Secondly, the results showed that EGF was effective in up-regulating mRNA expression of growth hormone (GH), somatolactin α (SLα) and somatolactin β (SLβ) via ErbB1 and ErbB2 and subsequently coupled to MEK1/2/ERK1/2 and PI3K/Akt/mTOR pathways, respectively. However, EGF was not effective in GH release in pituitary cells. Thirdly, we found that EGF strongly induced pituitary prolactin (PRL) release and mRNA expression, which was mediated by ErbB1 and subsequent stimulation of MEK1/2/ERK1/2 and PI3K/Akt/mTOR pathways. Interestingly, subsequent study further found that neurokinin B (NKB) significantly suppressed EGF-induced PRL mRNA expression, which was mediated by neurokinin receptor (NK2R) and coupled to AC/cAMP/PKA signal pathway. These results suggested that EGF could differently regulate the pituitary hormones expression in grass carp pituitary cells.

## 1. Introduction

The pituitary is an important endocrine gland, that secrets multiple important pituitary hormones including growth hormone (GH), prolactin (PRL), somatolactin alpha /beta (SLα/β), luteinizing hormone (LH) and follicle-stimulating hormone (FSH) [1,2]. These pituitary hormones are responsible for the process of endocrine regulation and metabolism, growth and reproduction development within the body [3,4]. Pituitary GH was involved in body growth [5] and metabolism [6]. In addition, it was also reported that GH played an important role in reproduction both in mammal and teleost [7,8]. Somatolactin is a fish specific pituitary hormone, which has two isoforms, namely SLα and SLβ, respectively [9]. Previous studies found that SLα and SLβ were involved in diverse functions in fish models, including melanosome aggregation [10,11], inflation of swim bladder during embryo development [12], reproduction [9], stress responses [13], lipid metabolism [12], and osmoregulation [14]. Gonadotropins (FSH/LH) are the most major mediator in brain-pituitary-gonad axis in vertebrates. In teleost, gonadotropins have been found to be involved in several reproductive functions, including in folliculogenesis, spermatogenesis, puberty onset, or gonadal differentiation [15]. PRL is also one of the most important pituitary hormones, which is known for its role in enabling female mammals to produce milk [16]. However, PRL was mainly involved in the osmoregulation in teleost [17].

In vertebrates, hypothalamic peptides have an effect on stimulating or inhibiting the production of pituitary hormones. Besides, these pituitary hormones are also regulated by several growth factors, which triggered cell responses such as cell survival, chemotaxis, proliferation and differentiation [18]. Up to now, epidermal growth factor (EGF) and insulin-like growth factors (IGFs) are the most extensively studied growth factors. The EGF ligand/receptor system was widely distributed in many tissues, such as skin, breast, liver, intestine, bone, gonad and central nervous system including pituitary, which identified as important molecules in mediating female reproduction, bone formation, epidermis, pathogenesis and progression of different carcinoma types [19,20]. In the pituitary, several studies have reported that IGFs were involved in the regulation of pituitary hormones in vertebrates. However, little is known about the pituitary hormone regulation by EGF. EGF is a 53-aa polypeptide, which activated its four specific receptors (ErbB1-4/Her1-4) [19] to stimulate several signal cascades, such as MEK/ERK and PI_3_K/Akt pathways and then modulate multiple cell processes including cell proliferation and survival [21,22]. In mammals, previous studies found that EGF induced GH and PRL secretion and mRNA expression in GH3 rat pituitary tumor cell lines [23,24]. In addition, EGF could also stimulate pituitary LH [25], thyroid-stimulating hormone (TSH) [26] and adrenocorticotrophic hormone (ACTH) release [27] in mammals. These results suggested that EGF might play an important role in growth and reproduction through modulating pituitary hormones secretion in mammal pituitaries. 

In zebrafish, previous study found that EGF had no effect on gonadotropins (FSHβ/LHβ) and GH mRNA expression in pituitary cells [28]. Besides, our recent study found that EGF induced SLα secretion and mRNA expression in grass carp pituitary [29]. However, the functional role of EGF in other pituitary hormones as well as the signal transduction is little known in teleosts. Therefore, in this study, using primary cultured pituitary cells as model, we investigated the pituitary functions of EGF on pituitary hormones in grass carp. Firstly, we examined the effect of EGF on FSHβ, LHβ, gonadotropin subunit alpha (GtHα), GH, PRL and SLα/SLβ secretion and mRNA expression in grass carp pituitary cells. Secondly, using pharmacological methods, we clarified the receptor specificity and signal transductions of EGF-induced pituitary hormones expression. Finally, we further studied the synergistic effect of NKB and EGF on PRL mRNA expression in grass carp pituitary cells.

## 2. Materials and Methods

### 2.1. Animals and Chemicals

About 18 months-old healthy grass carps (1+) (*Ctenopharyngodon idellus*) with a body weight (BW) of 1.0–1.5 kg were purchased from nearby markets and kept in the aquaria at 20 ± 2 °C for two weeks before used in the experiment. Since the grass carps at this stage were prepubertal and sexual dimorphism was not apparent, fish of mixed sexes were then used for pituitary cells preparation. In this process, grass carps were anesthetized in 0.05% tricaine methasulphonate (MS222) (Sigma, St. Louis, MO, USA) followed by spinosectomy according to the animal use regulations of Huazhong Agricultural University (Ethical Approval No. HBAC20091138; Date: 15 November 2009). Human EGF was acquired from GenScript Corporation (Nanjing, China), and before use in experiments, the EGF was dissolved in double-distilled deionized water and stored at −80 °C. Grass carp neurokinin B (NKB) was synthesized by GenScript Corporation (Nanjing, China) and subsequently diluted in DMSO with 1mM stocks and stored frozen at −80 °C. Other receptor specificity and post-receptor signal transduction of inhibitors (listed in Table S1) were diluted in DMSO as 10 mM frozen stocks. The final dilutions of DMSO were always ≤0.1% and did not affect pituitary hormone secretion/gene expression in our cell culture system [30]. Before drug treatment, these pharmacological agents were diluted with pre-warmed culture medium to appropriate concentrations for 15 min. At present study, all the cell culture medium we used was EGF free.

### 2.2. Cell Culture, RNA Isolation, Reverse Transcription and Real-Time PCR

Grass carp pituitaries were excised and washed in Hanks’ Balanced Salt Solution three times, and dispersed by trypsin/DNase II digestion method [31] as described previously. After that, grass carp pituitary cells were seeded in 24-well culture plates with precoated poly-d-lysine (Sigma-Aldrich, St. Louis, MO, USA) at a density of 2.5 × 10^6^ cells/well/mL for 2~3 h in plating medium, which contains 9.6 g MEM (Gibco, Waltham, MA, USA), 2.2 g NaHCO_3_, 6 g HEPES, 0.03 g penicillin, 0.05 g streptomycin in 1 L of ultrapure water. And then 5% FBS (Gibco, Waltham, MA, USA) was added in each well and continued incubating at 28 °C under 5% CO_2_ in plating medium for about 15 h. Finally, the plating medium was discarded and added by testing medium with EGF, which contains 9.6 g MEM (Gibco, Waltham, MA, USA), 2.2 g NaHCO_3_, 6 g HEPES, 1 g BSA and 0.03 g penicillin, 0.05 g streptomycin in 1 L of ultrapure water and continued to incubate for 48 h. After drug treatment, total RNA was extracted from individual well by adding Trizol reagent (500 μL/well) (Invitrogen, Carlsbad, CA, USA) and shaking the plate on the Orbital Shaker (Qilinbeler, Jiangsu, China) at 160–170 rpm/min for 10 min. Then total RNA was reversely transcribed by HifairTM III 1st Strand cDNA Synthesis Kit (gDNA digester plus) (Yeasen Biotech Co. Ltd., Shanghai, China) according to the manufacturer’s instructions. After RNA was isolated and reverse transcribed, the RT samples were used to detect the transcript levels of PRL, GH, SLα, SLβ, GtHα, FSHβ and LHβ by using ABI 7500 real-time PCR system with specific primers, respectively (See Table S2 for primer sequences and PCR condition). For real-time PCR, serial dilutions of plasmid DNA containing coding sequences for grass carp PRL, GH, SLα/SLβ, GtHα, and FSHβ/LHβ were used as the standard for data calibration. The PCR conditions were 30 s at 94 °C, 30 s at 58–60 °C, and 30 s at 72 °C, 20 s at 80 °C for a signal detection with total 40 PCR cycles. The specificity of qPCR reaction was confirmed by a melt curve analysis at the end of the reaction. And β-actin was conducted to the individual experiment to serve as the internal control for parallel real-time PCR measurement.

### 2.3. Measurement of PRL, GH and LH Secretion by Fluorescence-Based ELISA

The grass carp pituitary cells were incubated with EGF. Then, after drug treatment, the culture medium was harvested for measurement of PRL, GH and LH release by using Fluorescence Immunoassay (FIA), respectively. In this experiment, the recombinant grass carp PRL, GH and LH protein were synthesized and used to produce the polyclonal antibodies, respectively. The recombinant proteins were biotinylated and used as the tracer for the respective assays. Costar 96-well black plate (Thermo Fisher, Waltham, MA, USA) was precoated with protein A (0.5 µg/mL), which was used to load the protein samples with tracer and antibody, respectively (For information of PRL, GH and LH antibody, please refer to Table S3). After overnight incubation at 4 °C, each well was rinsed three times with washing buffer to clear non-specific binding of primary antibody. Then, the plate was continued to incubate for another 1 h with HRP-conjugate streptavidin (0.5 µg/mL) at 26 ± 2 °C. Subsequently, unbound second antibody was eliminated by decanting. Then, QuantaBlu^TM^ Fluorogenic Peroxidase Substrate (Thermo Scientific, Rockford, IL, USA) was added into individual wells for signal development. Finally, fluorescence signals were routinely detected by using a FluoStar OPTIMA-Fluorecence plate reader (BMG Labtech GmbH, Ortenberg, Germany).

### 2.4. Data Transformation and Statistical Analysis

In this experiment, for the measurement of PRL, GH and LH release, standard urves with a range from 0.98 to 500 ng/mL for target hormones were used for data calibration with a four-parameter logistic regression modal of GraphPad Prism 6 program (GraphPad, San Diego, CA, USA). For PRL, GH, SLα, SLβ, GtHα, FSHβ and LHβ mRNA expression, standard curves with a dynamic range of ≥10^5^ and a correlation coefficient of ≥0.95 were used for data calibration with ABI7500 software (Applied Biosystems, Foster City, MA, USA). PRL, GH, SLα, SLβ, GtHα, FSHβ and LHβ mRNA expression data were normalized with β-actin transcript level, and then the data as well as protein data were transformed as a percentage of the mean value in the control group without drug treatment (as “%Ctrl”). At present study, the data presented (as mean ± SEM) were pooled results from four separate replicates. All the data were analyzed with one-way ANOVA or two-way ANOVA by Dunnett’s test using Prism 6.0. Differences between experimental groups were regarded as significant at *p* < 0.05.

## 3. Results

### 3.1. EGF Reduced LHβ mRNA Expression in Grass Carp Pituitary Cells

**Figure 1:**
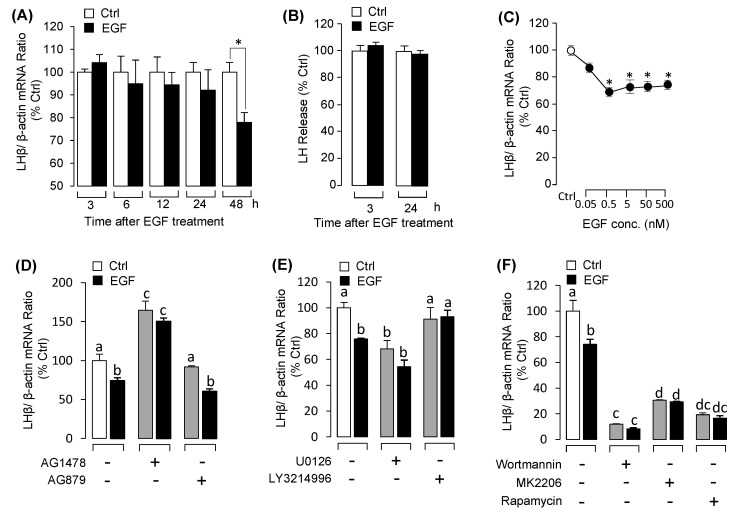
The effects of EGF on luteinizing hormone beta (LHβ) secretion and mRNA expression in grass carp pituitary cells: receptor specificity and signal pathways. (**A**) Time course of EGF (50 nM) on LHβ mRNA expression. (**B**) Time course of EGF (50 nM) on LH release in grass carp pituitary cells. (**C**) Dose experiment with increasing concentration of EGF (0.05–500 nM) on LHβ mRNA expression. for 48-h treatment. (**D**) The effects of EGF receptor inhibitors including ErbB1 inhibitor AG1478 (5 μM) and ErbB2 inhibitor AG879 (5 μM) on LHβ mRNA expression for 48 h. (**E**) The effects of 48-h cotreatment with MEK_1/2_ inhibitor U0126 (5 μM) or ERK_1/2_ inhibitor LY3214996 (5 μM) on EGF (50 nM)-reduced LHβ mRNA expression. (**F**) The effects of 48-h cotreatment with signal transduction inhibitors (5 μM) for PI3K/Akt/mTOR pathway on EGF (50 nM)-reduced LHβ mRNA expression. After drug treatment, the total RNA was collected and prepared for LHβ mRNA expression by using real-time PCR. Data presented are expressed as mean ± SEM (*n* = 4). *p <* 0.05 (“*”) was used to present significant differences among each group. The different letters represent a significant difference at *p* < 0.05 between groups (ANOVA followed by a Dunnett test).

**Figure 2:**
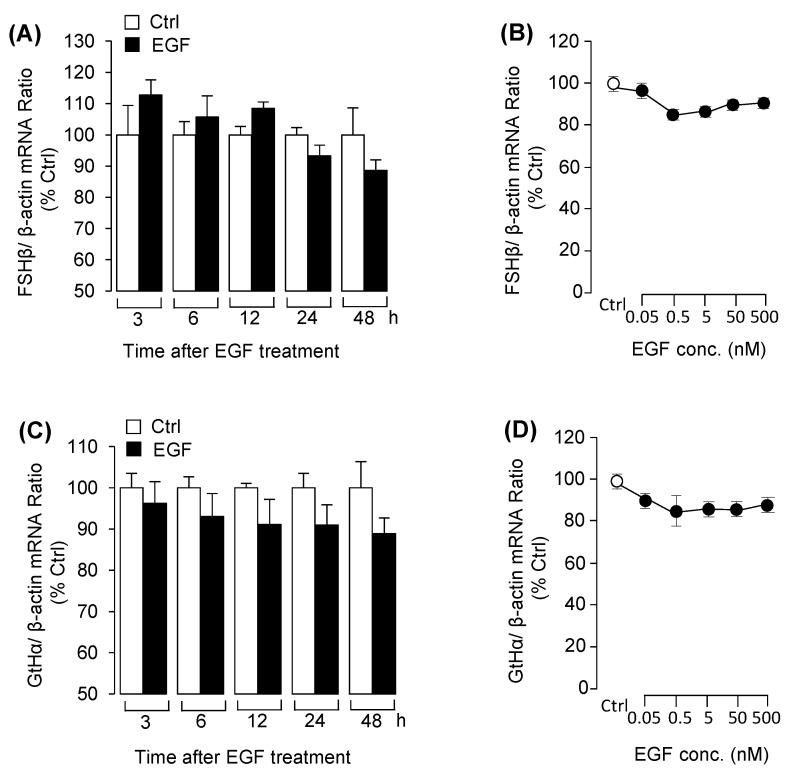
The effects of EGF on follicle-stimulating hormone beta (FSHβ) and gonadotropin subunit alpha (GtHα) mRNA expression in pituitary cells. (**A**) Time experiment of EGF (50 nM) on FSHβ mRNA expression from 3 to 48 h. (**B**) Dose experiment with increasing dose of EGF (0.05–500 nM) on FSHβ mRNA expression for 48-h incubation. (**C**) Time course of EGF (50 nM) on GtHα mRNA expression. (**D**) With increasing concentration of EGF (0.05–500 nM) on GtHα mRNA expression for 48-h incubation. After drug treatment, the total RNA was collected and prepared for FSHβ and GtHα mRNA expression by using real-time PCR. Data presented are expressed as mean ± SEM (*n* = 4).

With the grass carp pituitary cell culture established, the time- and dose-experiments were carried for examining the effect of EGF on the expression of LHβ, FSHβ and GtHα. The results revealed that EGF significantly reduced LHβ mRNA expression from 24 h to 48 h (Figure 1A). In the dose-dependent studies, incubation with increasing concentration of EGF (0.05–500 nM) for 48 h also down-regulated LHβ mRNA expression in a dose-related fashion (Figure 1C). However, EGF had no effect on LH release at the protein level in grass carp pituitary cells (Figure 1B). After that, using pharmacological approach to further elucidate the receptor specificity for LHβ regulation by EGF. The result showed that the inhibitory effect of EGF on LHβ mRNA expression was eliminated by simultaneous treatment with the ErbB1 inhibitor AG1478, but the ErbB2 inhibitor AG879 was not effective in this regard (Figure 1D). By using several pharmacological inhibitors targeting different signal pathways, we further examined the signal transduction mechanisms for EGF-reduced LHβ mRNA expression. As shown in Figure 1E, EGF (50 nM)-reduced LHβ mRNA expression was only reverted by simultaneous treatment with the ERK_1/2_ inhibitor LY3214996, but not MEK_1/2_ inhibitor U0126. In addition, treatment with wortmannin, MK2206 and rapamycin targeting for PI_3_K/Akt/mTOR pathway were not involved in the inhibitory effects of EGF on LHβ mRNA expression (Figure 1F). However, treatment of the grass carp pituitary cells with EGF for 48h had no effect on FSHβ and GtHα mRNA expression in the time- and dose-dependent experiment (Figure 2). In addition, to confirm that MEK/ERK and PI3K/AKT/mTOR cascades were involved in EGF-induced post-receptor signaling, the effects of EGF on ERK phosphorylation and AKT phosphorylation were tested in grass carp pituitary cells. As shown in Figure S1. These results suggested that MEK/ERK and PI3K/AKT/mTOR pathways could be involved in EGF-induced post receptor signal pathways.

### 3.2. EGF Induced Pituitary GH mRNA Expression, But No Effect on GH Secretion

**Figure 3:**
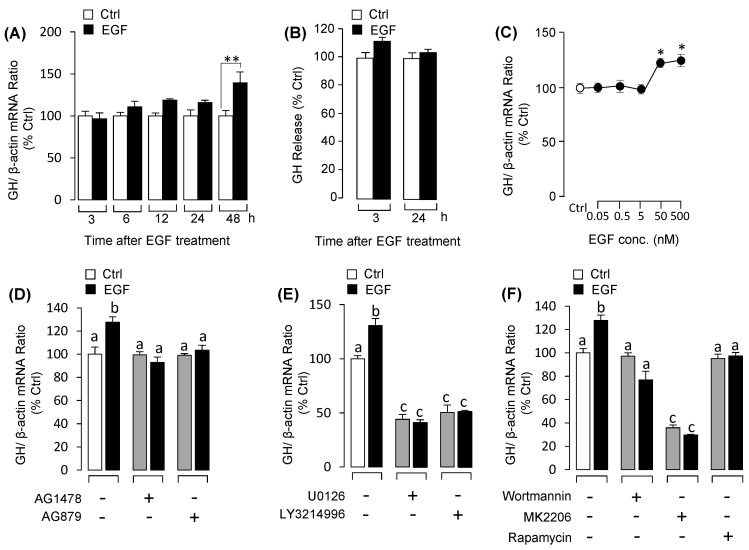
The effects of EGF on growth hormone (GH) secretion and mRNA expression in grass carp pituitary cells: receptor specificity and signal pathways. (**A**) Time course of EGF (50 nM) on GH mRNA expression. (**B**) Time course of EGF (50nM) on GH release in grass carp pituitary cells. (**C**) Dose-dependence of 48-h incubation with increasing levels of EGF (0.05–500 nM) on GH mRNA expression. (**D**) Receptor specificity for GH regulation by EGF. In this experiment, grass carp pituitary cells were treated for 48-h with EGF (50 nM) in the presence or absence of ErbB1 inhibitor AG1478 (5 μM) or ErbB2 inhibitor AG879 (5 μM), respectively. (**E**) The effects of 48-h cotreatment with MEK_1/2_ inhibitor U0126 (5 μM) or ERK_1/2_ inhibitor LY3214996 (5 μM) on EGF (50 nM)-induced GH mRNA expression. (**F**) The effects of 48-h cotreatment with PI_3_K inhibitor wortmannin (5 μM), Akt inhibitor MK2206 (5 μM) or mTOR inhibitor rapamycin (5 μM) on EGF (50 nM)-induced GH mRNA expression. After drug treatment, the total RNA was collected and prepared for real-time PCR of GH mRNA expression. Data presented are expressed as mean ± SEM (*n* = 4). *p <* 0.05 (“*”) and *p <* 0.01 (“**”) were used to present significant differences among each group. And the different letters represent a significant difference at *p* < 0.05 between groups (ANOVA followed by a Dunnett test).

To investigate the pituitary functions of EGF in pituitary GH, the primary cultured grass carp pituitary cells were challenged with EGF. As shown in Figure 3A, the time-course experiment indicated that EGF significantly elevated the transcript level of GH at 48 h. Following the time-course experiment, 48 h incubation with increasing levels of EGF (50–500 nM) significantly stimulated GH mRNA expression in a dose-dependent manner (Figure 3C). However, the fluorescence-based ELISA results showed that EGF failed to stimulate GH release in pituitary cells (Figure 3B). To further clarify the receptor specificity of EGF-induced GH mRNA expression, grass carp pituitary cells were challenged with EGF receptor inhibitors. As shown in Figure 3D, EGF induced GH mRNA expression at 48 h, and these stimulatory effects were abolished by both the ErbB1 inhibitor AG1478 and ErbB2 inhibitor AG879. For signal transduction, pituitary cells were co-treated with EGF (50 nM) in a presence of several inhibitors targeting different signal pathways for 48 h. As shown in Figure 3E, the up-regulation of GH mRNA expression effects induced by EGF were fully or partially blocked by MEK_1/2_ inhibitor U0126 and ERK_1/2_ inhibitor LY3214996 as well as PI_3_K inhibitor wortmannin, Akt inhibitor MK2206 or mTOR inhibitor rapamycin in pituitary cells (Figure 3F). 

### 3.3. EGF Stimulated Both SLα and SLβ mRNA Expression in Grass Carp Pituitary Cells

**Figure 4:**
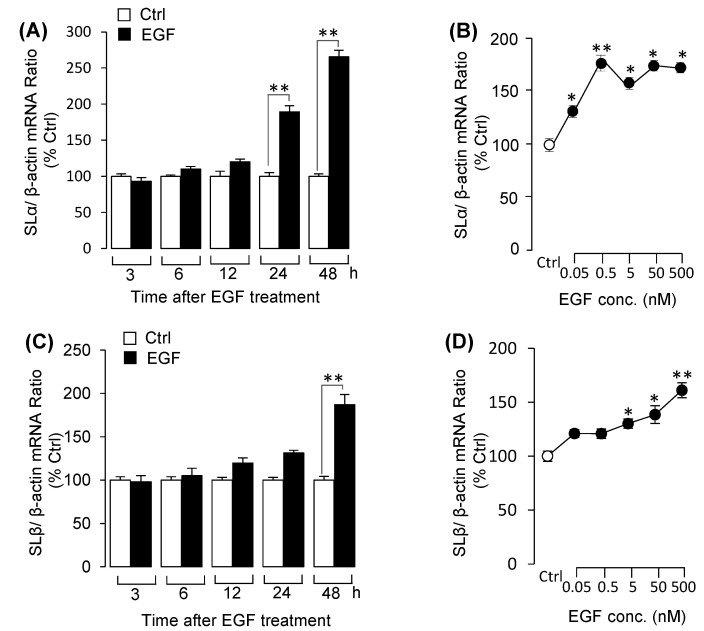
EGF induced somatolactin alpha (SLα) and somatolactin beta (SLβ) mRNA expression in pituitary cells. (**A**) Time experiment of EGF (50 nM) on SLα mRNA expression from 3 to 48 h. (**B**) Dose experiment with increasing dose of EGF (0.05–500 nM) on SLα mRNA expression for 48-h incubation. (**C**) Time experiment of EGF (50 nM) on SLβ mRNA expression. (**D**) Dose experiment with increasing levels of EGF (0.05–500 nM) on SLβ mRNA expression for 48-h incubation. After drug treatment, the total RNA was collected and prepared for SLα and SLβ mRNA expression by using real-time PCR. Data presented are expressed as mean ± SEM (*n* = 4). *p <* 0.05 (“*”) and *p <* 0.01 (“**”) were used to present significant differences among each group, (ANOVA followed by a Dunnett test).

**Figure 5:**
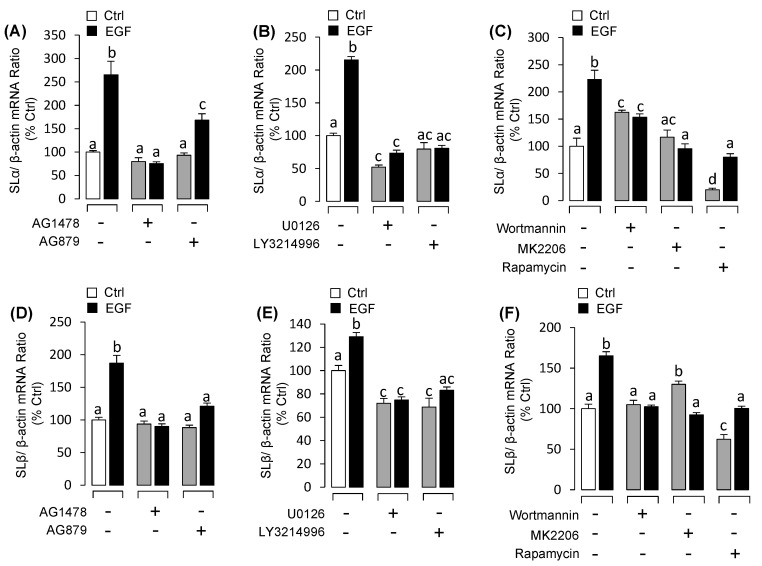
Receptor specificity and post-receptor signal pathways of EGF-induced SLα and SLβ mRNA expression. Receptor specificity for SLα (**A**) and SLβ (**D**) regulation by EGF. In this experiment, grass carp pituitary cells were treated for 48-h with EGF (50 nM) in the presence or absence of ErbB1 inhibitor AG1478 (5 μM) or ErbB2 inhibitor AG879 (5 μM). The effects of 48-h co-treatment with MEK_1/2_ inhibitor U0126 (5 μM) or ERK_1/2_ inhibitor LY3214996 (5 μM) on EGF (50 nM)-induced SLα (**B**) and SLβ (**E**) mRNA expression, respectively. The effects of 48-h co-treatment with PI_3_K inhibitor wortmannin (5 μM), Akt inhibitor MK2206 (5 μM) or mTOR inhibitor rapamycin (5 μM) on EGF (50 nM)-induced SLα (**C**) and SLβ (**F**) mRNA expression, respectively. After drug treatment, the total RNA was collected and prepared for real-time PCR of SLα and SLβ mRNA expression. Data presented are expressed as mean ± SEM (*n* = 4). The different letters represent a significant difference at *p* < 0.05 between groups (ANOVA followed by a Dunnett test).

Similarly, using pituitary cells as model, we detected the effects of EGF on SLα and SLβ mRNA expression at pituitary level. Time-course experiments indicated that EGF significantly enhanced both SLα (Figure 4A) and SLβ (Figure 4C) mRNA expression in a time-related fashion. Following the time-course experiment, 48h incubation with the increasing concentrations of EGF could significantly stimulate both SLα (Figure 4B) and SLβ (Figure 4D) mRNA expression in a dose-dependent manner. Using pharmacological approach again to elucidate the receptor specificity for EGF-induced somatolactin expression. The results showed that EGF-induced SLα (Figure 5A) and SLβ (Figure 5D) mRNA expression were blocked by both ErbB1 inhibitor AG1478 and ErbB2 inhibitor AG879. To further study the signal transductions, grass carp pituitary cells were exposed to EGF combination with/without various inhibitors targeting the various signal pathways. The results revealed that EGF-induced SLα (Figure 5B) and SLβ (Figure 5E) mRNA expression were both significantly abolished by simultaneous incubation with MEK_1/2_/ ERK_1/2_ pathway inhibitors U0126 and LY3214996, respectively. In addition, PI_3_K inhibitor wortmannin, Akt inhibitor MK2206 or mTOR inhibitor rapamycin could also block EGF-induced SLα (Figure 5C) and SLβ (Figure 5F) mRNA expression in grass carp pituitary cells, respectively. 

### 3.4. EGF Strongly Induced PRL Secretion and mRNA Expression in Grass Carp Pituitary Cells

**Figure 6:**
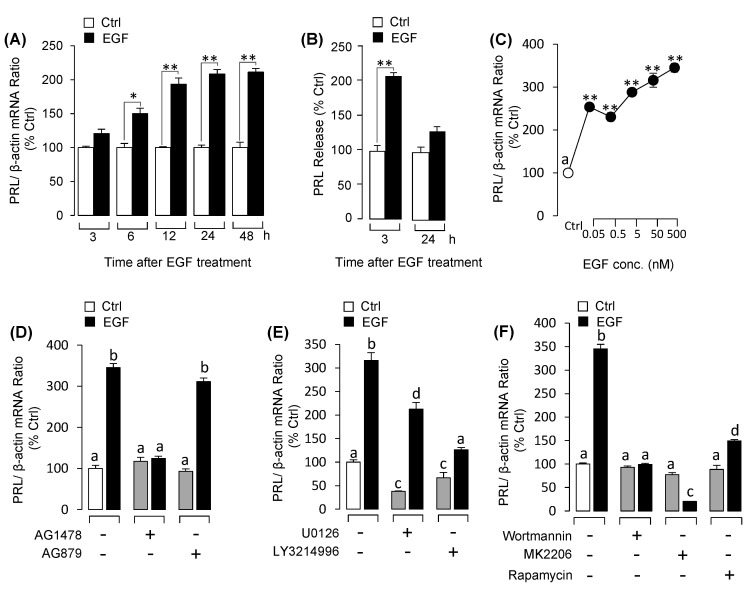
EGF induced prolactin (PRL) mRNA expression in grass carp pituitary cells: Receptor specificity and signal pathways. (**A**) Time course of EGF (50 nM) on PRL mRNA expression. (**B**) Time course of EGF (50 nM) on PRL release in grass carp pituitary cells. (**C**) Dose-dependence of 48-h treatment with increasing levels of EGF (0.05–500 nM) on PRL mRNA expression. (**D**) Receptor specificity for PRL regulation by EGF. In this experiment, grass carp pituitary cells were treated for 48-h with EGF (50 nM) in the presence or absence of ErbB1 inhibitor AG1478 (5 μM) or ErbB2 inhibitor AG879 (5 μM). (**E**) The effects of 48-h cotreatment with MEK_1/2_ inhibitor U0126 (5 μM) or ERK_1/2_ inhibitor LY3214996 (5 μM) on EGF (50 nM)-induced PRL mRNA expression. (**F**) The effects of 48-h cotreatment with PI_3_K inhibitor wortmannin (5 μM), Akt inhibitor MK2206 (5 μM) or mTOR inhibitor rapamycin (5 μM) on EGF (50 nM)-induced PRL mRNA expression. After drug treatment, the total RNA was collected and prepared for real-time PCR of PRL mRNA expression. Data presented are expressed as mean ± SEM (*n* = 4). *p <* 0.05 (“*”) and *p <* 0.01 (“**”) were used to present significant differences among each group. And the different letters represent a significant difference at *p* < 0.05 between groups (ANOVA followed by a Dunnett test).

Treatment of grass carp pituitary cells with EGF (50 nm) for 48 h significantly increased the expression levels of PRL in time-dependent manner (Figure 6A). For the dose-dependent experiment, 48h incubation with the increasing concentrations of EGF (0.05–500 nM) also significantly induced PRL mRNA expression in a dose-dependent manner (Figure 6C). In addition to the mRNA level, the protein levels of PRL were also detected. The results in Figure 6B showed that 3 h incubation with EGF significantly induced PRL release in grass carp pituitary cells, whereas 24h had no effect on PRL release. To further elucidate the receptor specificity for EGF-induced PRL mRNA expression, EGF receptor antagonists were used to co-treat with EGF. The results showed that the up-regulation effects of EGF on PRL mRNA expression substantially suppressed by ErbB1 inhibitor AG1478 (Figure 6D), but ErbB2 inhibitor AG879 was not involved (Figure 6D). To elucidate the signal pathways for PRL regulation by EGF, pituitary cells were co-treated for 48 h with signal transduction inhibitors targeting MEK_1/2_/ERK_1/2_, and PI_3_K/Akt/mTOR pathways. As shown in Figure 6E, EGF-induced PRL mRNA expression was significantly inhibited by MEK_1/2_/ERK_1/2_ pathway inhibitors U0126 and LY3214996, respectively as well as PI_3_K/Akt/mTOR pathways inhibitors wortmannin, MK2206 and rapamycin (Figure 6F).

### 3.5. NKB Suppressed EGF-Induced PRL mRNA Expression in Grass Carp Pituitary Cells

**Figure 7:**
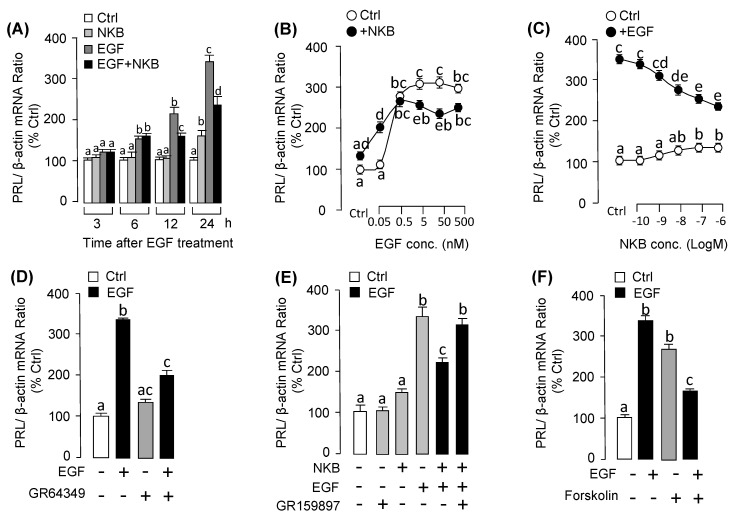
The effect of EGF and NKB on PRL mRNA expression in grass carp pituitary cells. (**A**) Time course of EGF (50 nM), NKB (1 µM), EGF (50 nM) + NKB (1 µM) on PRL mRNA expression. (**B**) Dose experiment of 24-h incubation with increasing dose of EGF (0.05–500 nM) on basal and NKB (1 µM)-induced PRL mRNA expression. And the transparent dotted boxes were to present different comparisons for each dose of EGF and NKB treatments. (**C**) Dose experiment of 24-h treatment with increasing concentration of NKB (0.1–1000 nM) on basal and EGF (50 nM)-induced PRL mRNA expression. The transparent dotted boxes were to present different comparisons for each dose of EGF and NKB treatments. (**D**) Effects of NK2R agonist GR64349 (10 μM) on EGF (50 nM)-induced PRL mRNA expression. (**E**) Effect of NK2R antagonist GR159897 (10 µM) on EGF (50 nM) + NKB (1 µM)-induced PRL mRNA expression. In this study, pituitary cells were treated for 24-h with NKB (1 µM), EGF (50 nM) and EGF (50 nM) + NKB (1 µM) in the presence or absence of NK2R antagonist GR159897 (10 µM). (**F**) Effects of AC activator Forskolin (1 μM) on EGF (50 nM)-induced PRL mRNA expression for 24 h. After drug treatment, the total RNA was collected and prepared for PRL mRNA expression by using real-time PCR. In this experiment, the one/two-way ANOVA was tested for significant differences among dose experiments with EGF and NKB treatments. Data presented are expressed as mean ± SEM (*n* = 4). The different letters represent a significant difference at *p* < 0.05 between groups (ANOVA followed by a Dunnett test).

To shed light on the synergistic action of EGF and NKB for PRL mRNA expression at pituitary level, primary cultured pituitary cells were incubated with EGF alone or co-treated with NKB. As shown in Figure 7A, EGF or NKB alone could both significantly induce PRL mRNA expression in the time-related fashion. Interestingly, the stimulatory effects of EGF on PRL mRNA expression was obviously suppressed by co-treatment with NKB after 12 h (Figure 7A). In the parallel dose-dependent experiment, the results showed that the effect of NKB in stimulating PRL mRNA was clearly enhanced with low dose of EGF (at 0.05 nM). Interestingly, EGF-induced PRL mRNA expression was decreased in the high doses of EGF (5–500 nM) (Figure 7B). To further elucidate this phenomenon, the dose experiment with increasing dose of NKB (0.1–1000 nM) was carried. As shown in Figure 7C, NKB-induced PRL mRNA expression was significantly declined in a dose-related manner with increasing levels of NKB (0.1–1000 nM). To understand the receptor specificity and signal transduction for this pituitary action, the pharmacological agonists and antagonists were used. As shown in Figure 7D, EGF-induced PRL mRNA expression was suppressed by NK2R agonist GR64349. In the parallel experiment, the inhibitory effect of NKB on EGF-induced PRL mRNA expression could be recovered by NK2R antagonist GR159897 (Figure 7E). Furthermore, AC activator forskolin could also mimic the inhibitory effect of NKB on EGF-induced PRL mRNA expression in grass carp pituitary cells (Figure 7F).

## 4. Discussion

In mammals, it is well recognized that the regulation of pituitary hormones biosynthesis and secretion by growth factors has gained increasing attention in recent years. However, the function of EGF in the regulation of pituitary hormones are little known in teleost. By using transcriptome analysis, our previous study found that EGF could differently regulate the pituitary hormones in grass carp pituitary cells at 24 h (Table S4) [32]. In the present experiment, using primary cultured grass carp pituitary cells as model, we further examined the effect of EGF on pituitary hormones (FSH, LH, GH, PRL and SLα/SLβ) secretion and mRNA expression in teleost.

It is known that gonad development in animals is controlled by pituitary gonadotropins, namely LH and FSH. Both LH and FSH are heterodimers, sharing a common alpha-subunit (GtHα) and differing in their beta-subunits (LHβ and FSHβ) [2,33]. In this study, we found that EGF significantly reduced LHβ mRNA expression coupled to ErbB1 via ERK_1/2_ pathway in grass carp pituitary cells. Interestingly, previous studies have been demonstrated that LH could induce the EGF-like growth factors expression to induce the oocyte maturation in rat ovary [34,35,36]. In teleost, it has also been reported that EGF induced oocyte maturation in zebrafish and goldfish [10]. These results indicated that EGF-reduced pituitary LHβ mRNA expression might be a negative feedback effect of ovary EGF induced by LH during gonadal development. In addition, it is confusion that EGF inhibited LHβ mRNA expression could not activate MEK1/2, we speculated that there would be other signal mechanisms to crosstalk with ERK1/2 at pituitary level. GH is the important somatotropin and highly expressed in the pituitary in vertebrate [4,37]. Our experiment demonstrated that EGF stimulated the GH expression. However, EGF had no effect on GH release, in contrast to its effect in mammals [23]. The difference effect of EGF on GH expression between mammals and teleost would be related to use of different approaches and different models. Somatolactin is a fish-specific somatotropin released from the posterior pituitary [37], which have two isoforms, namely SLα and SLβ [9,30,38]. In teleost, our recent study found that EGF significantly induced SLα release and mRNA expression in grass carp pituitary cells [29]. In this study, we further demonstrated that EGF could also have an effect on up-regulating the SLβ mRNA expression. In addition, we firstly demonstrated the signal transductions of EGF-induced GH and SLβ mRNA, which were both coupled to ErbB1 and ErbB2 via MEK_1/2_/ERK_1/2_ and PI_3_K/Akt/mTOR pathway in grass carp pituitary.

In mammals, early studies have reported that EGF could stimulate PRL release and mRNA expression in the pituitary [39]. Similarly, in our study, we found that EGF significantly promoted pituitary PRL release and mRNA expression in teleost. In mammals, it is well-known that PRL could promote milk production [40]. Interestingly, recent study found that the primary function of PRL is osmoregulation via governing the uptake and homeostasis of Na^+^, K^+^ and Cl^−^ in teleost [16,41]. Therefore, we speculated that EGF might play a role in osmotic regulation in teleost. In addition, the stimulatory effect of EGF on PRL mRNA expression was blocked by ErbB1 inhibitor but not ErbB2 inhibitor. These results were agreement with the previous reports that EGF-induced PRL gene expression via ErbB1 but not ErbB2 in GH3 cells [42]. 

Neurokinin B (NKB) is one member of tachykinin peptide family, which played an important role in the regulation of puberty onset [43]. Similar to EGF, NKB could also significantly induce PRL release and mRNA expression in pituitary cells [44]. Interestingly, EGF-induced pituitary PRL mRNA expression was suppressed with the increasing concentrations of NKB. However, the effect of NKB in stimulating PRL mRNA was enhanced with low dose of EGF (at 0.05 nM). The reason for this phenomenon is unknow, we speculated that it would be related to the function role of RPL in osmoregulation, whether the homeostasis of osmoregulation would be broken down when the PRL highly expressed in the teleost. Further studies found that the inhibitory effect of NKB on EGF-induced PRL mRNA expression was mimicked by NK2R agonist and recovered by NK2R antagonist. In addition, AC activator forskolin could also mimic the inhibitory effect of NKB on EGF-induced PRL mRNA expression in grass carp pituitary. These results suggested that the inhibitory effect of NKB on EGF-induced pituitary PRL mRNA expression was mediated by NK2R coupled to AC/cAMP/PKA pathway. As we know, NK2R is one member of G protein-coupled receptors (GPCRs), and EGFR is the typical receptor-tyrosine kinases (RTKs) in the cell surface. Previous studies have found that GPCRs could cross-talk with EGFR in cancer cell lines [45]. Therefore, we speculated that the inhibition effect of EGF and NKB on PRL mRNA expression was mediated by a negative cross-talk of post-receptor signaling between EGFR and NK2R in grass carp pituitary.

**Figure 8:**
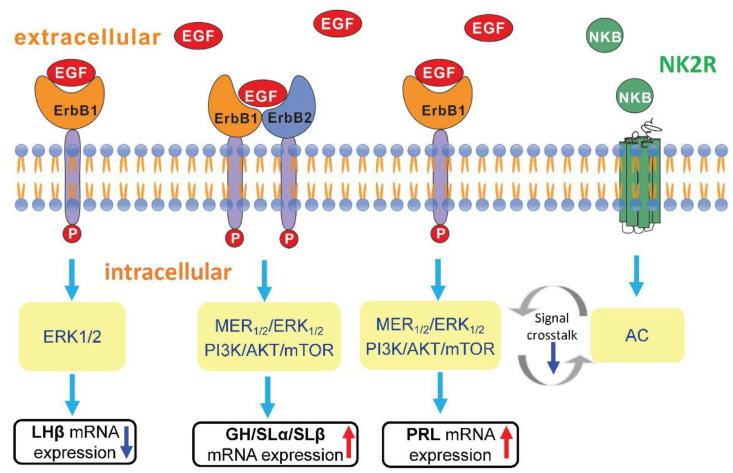
Working modal of pituitary hormones regulation by EGF in grass carp pituitary cells. EGF-reduced LHβ mRNA expression was mediated by ErbB1 homodimerization coupled to ERK_1/2_ pathway. EGF-induced GH, SLα and SLβ mRNA expression were mediated by ErbB1 and ErbB2 heterodimerization coupled to MEK_1/2_/ERK_1/2_ pathway and PI_3_K/Akt/mTOR pathway, respectively. EGF-induced PRL release and mRNA expression was mediated by ErbB1 via MEK_1/2_/ERK_1/2_ pathway and PI_3_K/Akt/mTOR pathway. In addition, the inhibitory effect of NKB on EGF-induced PRL mRNA expression was mediated by NK2R coupled to AC/cAMP/PKA pathway. The blue down arrows were represented by down-regulation and the red up arrows were represented by up-regulation. The grey circle arrows were indicated that the hypothesis of cross-talk among signal pathways.

In summary, the effects of EGF on pituitary hormones release and mRNA expression were detected in grass carp pituitary cells. The results showed that EGF significantly reduced LHβ mRNA expression via ErbB1 and subsequent stimulation of ERK_1/2_ pathway only. However, EGF have no effect on pituitary GtHα and FSHβ mRNA expression. In addition, EGF could induce GH, SLα and SLβ mRNA expression couple to ErbB1 and ErbB2 via MEK_1/2_/ERK_1/2_ and PI_3_K/Akt/mTOR pathways in grass carp pituitary cells, respectively. In the parallel study, we also confirmed that EGF significantly induced pituitary PRL release and mRNA expression, which was mediated by ErbB1 but not ErbB2 and subsequent stimulation of MEK_1/2_/ERK_1/2_ and PI_3_K/Akt/mTOR pathways. Further study found that the stimulatory effect of EGF-induced PRL mRNA expression was suppressed by co-treatment with NKB and mediated by NK2R via AC/cAMP/PKA pathways in grass carp pituitary cells (Figure 8). The effect of EGF and NKB on pituitary PRL mRNA expression might be mediated by the cross-talk between EGFR and NK2R. Taken together, these results demonstrated that EGF could differently regulate the gonadotropins, growth hormone, prolactin and somatolactins expression in grass carp pituitary level.

## 5. Conclusions

In conclusion, using grass carp pituitary cells as model, we investigated the direct pituitary actions of EGF in teleost. The results showed that EGF could reduce LHβ mRNA expression, but no effect on LH release. In addition, EGF could significantly induce GH, PRL, SLα and SLβ mRNA expression in grass carp pituitary cells. Interestingly, EGF-induced pituitary PRL mRNA expression could be significantly suppressed by NKB cotreatment. These results demonstrated that EGF could differently regulate the pituitary hormones expression in teleost. 

## Supplementary Materials

Supplementary materials can be found at https://www.mdpi.com/2079-7737/9/9/279/s1. Table S1: The information for the drugs used in receptor specificity and signal transduction. Table S2: Primers used for quantitative real-time PCR in this study. Table S3: Antibodies used in fluorescence immunoassay. Table S4: The effects of EGF on pituitary hormones at 24 h by transcriptome analysis. Fragments per kilobase of exon per million fragments mapped (FPKM), Fold Change (FC). Figure S1: EGF-induced protein phosphorylation of ERK and AKT. The grass carp pituitary cells were treated with 50 nM EGF for 30 min. After that, cell lysate was prepared for Western blot by using the antibody for phosphorylated ERK and total ERK (A) and phosphorylated AKT and total AKT (B), respectively. Parallel blotting of β-actin was used as an internal control.

## Abbreviations

AC	adenylate cyclase
Akt	protein kinase B
cAMP	cyclic adenosine monophosphate
EGF	epidermal growth factor
EGFR	epidermal growth factor receptor
ERK	extracellular signal-regulated kinase
FSH	follicle-stimulating hormone
GH	growth hormone
GPCR	G protein coupled receptor
GtHα	gonadotropin subunit alpha
HB-EGF	Heparin-binding EGF-like growth factor
LH	luteinizing hormone
MAPK	mitogen-activated protein kinase
mTOR	mammalian target of rapamycin
NKB	neurokinin B
NKR	neurokinin receptor
PI3K	phosphatidylinositol-3-kinase
PKA	protein kinase A
PRL	prolactin
PRL	prolactin
SLα	somatolactin α
SLβ	somatolactin β
TAC3	tachykinin 3
