# Supplementary material for: Pituitary Actions of EGF on Gonadotropins, Growth Hormone, Prolactin and Somatolactins in Grass Carp
Source: Biology (Basel). 2020 Sep 8;9(9):279. doi: 10.3390/biology9090279 (PMC7564354; doi:10.3390/biology9090279)
Supplement: Supplementary file 1 [file biology-09-00279-s001.pdf]

Supplemental Methods

Experiment 1#: Receptor specificity of EGF-induced/reduced GH, PRL, SLα/SLβ and LHβ mRNA expression in grass carp pituitary cells.

In this experiment, grass carp pituitary cells were incubated with EGF (50 nM), AG1478 (5 μM), EGF (50nM) + AG1478 (5 μM), AG879 (5μM), or EGF (50nM) + AG879 (5 μM), respectively. For the cotreatment of EGF and EGFR inhibitors, grass carp pituitary cells were firstly pretreated with EGF receptor inhibitor AG1478 (5 μM) or AG879 (5 μM) for 30 min, respectively. Then, grass carp pituitary cells were treated with EGF (50 nm) together. After 48 h treatment, the cells were collected with 500 μL/well Trizol (Invitrogen, Carlsbad, CA, USA) to extract the total RNA and reversely transcribed by HifairTM III 1st Strand cDNA Synthesis Kit (gDNA digester plus) (Yeasen Biotech, Shanghai, China). Finally, the RT samples were subject to qPCR using ABI 7500 real time PCR system (Applied Biosystems, USA) to detect the mRNA expression of grass carp GH, PRL, SLα/SLβ and LHβ with specific primer, respectively.

Experiment 2#: Signal transduction of EGF-induced/reduced GH, PRL, SLα/SLβ and LHβ mRNA expression in grass carp pituitary cells.

In the experiment, similarly, the pharmacological blockers targeting different signal pathways, including PI3K/AKT/mTOR pathway and MEK1/2/ERK1/2 pathway were pretreated with grass carp pituitary cells for 30 min, respectively. And then, each well was added the EGF (50 nM) to treat the grass carp pituitary cells together. After 48h treatment, total RNA was extracted from each well by Trizol reagent and reversely transcribed by HifairTM III 1st Strand cDNA Synthesis Kit (gDNA digester plus) (Yeasen Biotech, Shanghai, China). Finally, ABI 7500 real-time PCR system was conducted to detect the mRNA transcription of GH, PRL, SLα/SLβ and LHβ with specific primers, respectively.

Experiment 3#: Western blot for signaling kinases

In this experiment, grass carp pituitary cells were treated with or without EGF for 30 min based on time course validation. After drug treatment, the cell medium was discarded from individual well and the pituitary cells were rinsed with PBS. The pituitary cells were lysed in RIPA buffer (50 mM Tris. HCl, 150 mM NaCl, 1 mM EDTA, 1% NP-40, and 0.25% Na deoxycholate) containing a final concentration of 1 × protease/phosphatase inhibitor cocktail (Roche). After that, the cells lysate was centrifuged by 12000×g at 4 ℃, and the supernatant were collected. Using the antibodies of p-ERK (1:5,000), t-ERK (1:1,000), p-AKT (1:1,000) and t-AKT (1:1,000) to detected the phosphorylation. The dilutions of these antibodies were recommended by the manufactures. After the incubation with antibodies respectively, the membranes were washed three times to remove non-specific binding of primary antibodies and the HRP-conjugated secondary antibodies [goat anti-rabbit IgG (1:5,000)] were used for signal development. Finally, using SuperSignal West Pico (PIERCE, Rockford) as the substrate and quantified using the IC440 CF Digital Science Image Station (Eastman Kodak) to detect the Chemiluminescence signals for target immune-reactivity. In these experiments, Western blot of β-actin was used as an internal control using its antibody (1:15,000; Oncogen, Cambrige, MA).

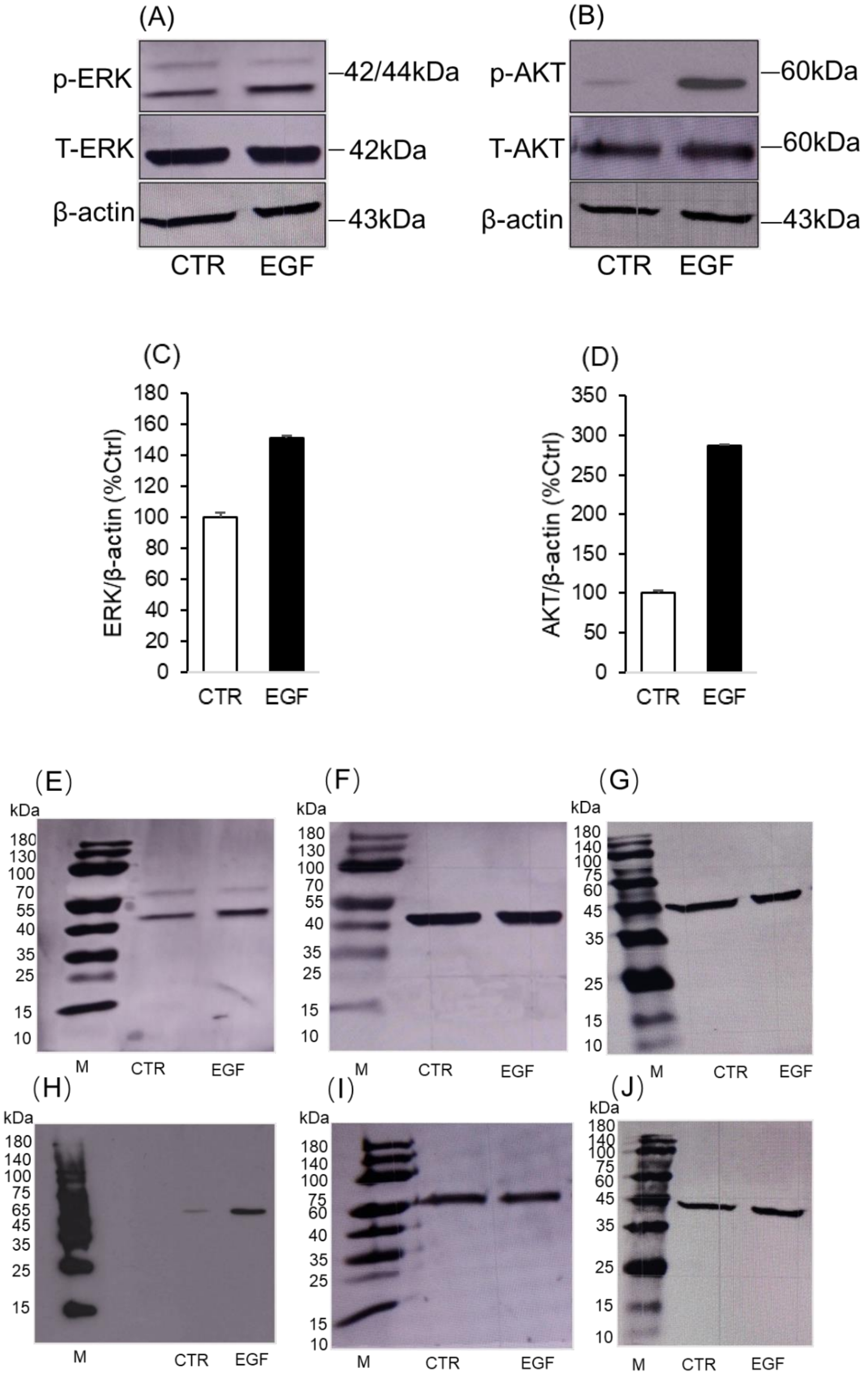

Figure S1. EGF-induced protein phosphorylation of ERK and AKT. The grass carp pituitary cells were treated with 50nM EGF for 30 min. After that, cell lysate was prepared for Western blot by using the antibody for phosphorylated ERK and total ERK (**A**) and phosphorylated AKT and total AKT (**B**), respectively. Parallel blotting of β-actin was used as an internal control. The quantified graph of ERK (**C**) and AKT (**D**) are the representative blots in each group. Data presented are expressed as mean ± SEM (n = 3). The groups denoted by different letters represent a significant difference at $P<0.05$. (**E–J**) The complete western blot figures of EGF-induced protein phosphorylation AKT and ERK.

Table S1. The information for the drugs used in receptor specificity and signal transduction.

Item	Function	Manufacture	Cat. No.
AG1478	ErbB1 inhibitor	MCE	HY-13524
AG879	ErbB2 inhibitor	MCE	HY-20878
U0126	MEK1/2 inhibitor	MCE	HY-12031
LY3214996	ERK1/2 inhibitor	MCE	HY-101494
Wortmannin	PI3K inhibitor	MCE	HY-10197
MK 2206	AKT inhibitor	MCE	HY-10358
Rapamycin	mTOR inhibitor	MCE	S1039
GR64349	NK2R agonist	Tocris Bioscience	1668
GR159897	NK2R antagonist	Tocris Bioscience	1274
Forskolin	AC activator	Sigma	F6886

Table S2. Primers used for quantitative real-time PCR.

qPCR Primers	Forward Primer	Reverse Primer	Length	Annealin Tm	Acc. No.
LHβ	ACATCCTCCTTCTCTTATTCTG	CAAGCGGACCGTCTCATAG	230	60℃	EF565171
FSHβ	TTCGTTGTTATGGTGATGCT	CGTGAAAACCGAGTCAGTCC	310	58℃	EF194762
GTHα	GATATGACTAACTTTGGATGTG	TAGTAACAGGTGCTACAGTGG	263	52℃	EU095936
GH	GTGGTGCTGGTTAGTTTGTTGG	CTTCAACATAGAGCTCTTCTG	252	54℃	AY616661
PRL	CTCAGCACCTCTCTCACCAATGACC	GCGGAAGCAGGACAACAGAAAATG	400	60℃	EU074210
SLα	ACCCACTGTACTTCAATCTCC	CGTCGTAACGATCAAGAGTAG	283	52℃	EF372074
SLβ	TGTTTGAGGAGATGCTCGTTT	CCACCGTCACCCAATATCTGT	289	52℃	EF372075
β-Actin	CTGGTATCGTGATGGACTCT	AGCTCATAGCTCTTCTCCAG	280	56℃	M25013

Table S3. Antibodies used in fluorescence immunoassay.

Protein Target	Antigen and Source of Sequence Information	Name of Antibody	Name of Individual Providing the Antibody	Species for Raising Antibody/Application
grass carp PRL	Recombinant grass carp PRL (GenBank EF565171)	grass carp PRL antibody	Dr. Hu GF, Huazhong Agricultural University	Polyclonal in Rabbit
grass carp GH	Recombinant grass carp GH (GenBank EF552359)	grass carp GH antibody	Dr. Hu GF, Huazhong Agricultural University	Polyclonal in Rabbit
grass carp LH	Recombinant grass carp LHβ (GenBank EF565171)	grass carp LH antibody	Dr. Hu GF, Huazhong Agricultural University	Polyclonal in Rabbit

Table S4. The effects of EGF on pituitary hormones at 24h by transcriptome analysis. Fragments per kilobase of exon per million fragments mapped (FPKM), Fold Change (FC).

Gene name	CTR_(fpkm)	EGF_(fpkm)	FC	*p*-value
GH	291581.5	267422.2	0.917144	5.89×10^{-1}
PRL	16916.15	31527.79	1.863769	1.09×10^{-51}
SLα	8960.87	16417.36	1.832117	2.80×10^{-49}
SLβ	792.78	818.17	1.032027	4.50×10^{-2}
GtHα	43405.87	37831.61	0.871578	9.36×10^{-2}
LHβ	813.94	746.03	0.916566	6.08×10^{-1}
FSHβ	236.6	214.39	0.906128	5.26E-01
